# Gastric Microenvironment—A Partnership between Innate Immunity and Gastric Microbiota Tricks *Helicobacter pylori*

**DOI:** 10.3390/jcm10153258

**Published:** 2021-07-23

**Authors:** Cristina Oana Mărginean, Lorena Elena Meliț, Maria Oana Săsăran

**Affiliations:** 1 Department of Pediatrics I, George Emil Palade University of Medicine, Pharmacy, Science, and Technology of Târgu Mureș, Gheorghe Marinescu Street No. 38, 540136 Târgu Mureș, Romania; marginean.oana@gmail.com; 2 Department of Pediatrics III, George Emil Palade University of Medicine, Pharmacy, Science, and Technology of Târgu Mureș, Gheorghe Marinescu Street No. 38, 540136 Târgu Mureș, Romania; oanam93@yahoo.com

**Keywords:** gastric microbiota, innate immunity, *Helicobacter pylori*, bacterial community, children

## Abstract

*Helicobacter pylori* (*H. pylori*) carcinogenicity depends on three major factors: bacterial virulence constituents, environmental factors and host’s genetic susceptibility. The relationship between microenvironmental factors and *H. pylori* virulence factors are incontestable. *H. pylori* infection has a major impact on both gastric and colonic microbiota. The presence of non-*H. pylori* bacteria within the gastric ecosystem is particularly important since they might persistently act as an antigenic stimulus or establish a partnership with *H. pylori* in order to augment the subsequent inflammatory responses. The gastric ecosystem, i.e., microbiota composition in children with *H. pylori* infection is dominated by *Streptoccocus*, *Neisseria*, *Rothia* and *Staphylococcus*. The impairment of this ecosystem enhances growth and invasion of different pathogenic bacteria, further impairing the balance between the immune system and mucosal barrier. Moreover, altered microbiota due to *H. pylori* infection is involved in increasing the gastric T regulatory cells response in children. Since gastric homeostasis is defined by the partnership between commensal bacteria and host’s immune system, this review is focused on how pathogen recognition through toll-like receptors (TLRs—an essential class of pathogen recognition receptors—PRRs) on the surface of macrophages and dendritic cells impact the immune response in the setting of *H. pylori* infection. Further studies are required for delineate precise role of bacterial community features and of immune system components.

## 1. Introduction

*Helicobacter pylori* (*H. pylori*) is probably the most common bacterial infection worldwide. As it is well-known, this infection is particularly important in children since it is usually acquired during childhood and its seroprevalence increases with age. Moreover, if left untreated, *H. pylori* infection results in a chronic inflammation of the gastric mucosa and imminent long-term carcinogenesis. Thus, it was proved that 80% of all gastric cancers and 5.5% of all malignancies are related to the injuries produced by this bacterium [1,2]. The World Health Organization considered *H. pylori* as a class I carcinogen since gastric cancers associated to this infection are amongst the first three most common causes of death worldwide [3]. Its carcinogenicity depends on three major factors: bacterial virulence constituents, environmental factors and host’s genetic susceptibility [4]. In terms of bacterial virulence, *H. pylori* has developed a wide spectrum of mechanisms in order to survive and escape the host’s defense immune responses. Among these mechanisms, we recall the ability to regulate T-cell responses, the inhibition of cathepsin X or the altered structure of lipopolysaccharides, which are definitely involved in the mediation of resistance against drugs [5]. Therefore, a recent study performed on European individuals revealed that the primary resistance rates against clarithromycin and metronidazole have reached 17.5% and 34.9%, respectively [6]. Moreover, the same study pointed out that resistant strains to levofloxacin have emerged impairing its effectiveness as rescue therapy [6]. As we already mentioned, environmental factors also play a major role in the persistence of this infection, including socio-economic conditions, originating area, dietary habits or certain practices within the household [7,8,9]. Gastric microenvironment defined mostly by gastric microbiota influences the development of *H. pylori* gastritis and even immune responses [10,11]. Thus, we hypothesize that the complex interrelationship between *H. pylori*, gastric microbiota and innate immune responses shapes the pathophysiology of *H. pylori*-related disorders and the persistence of this infection during adulthood.

## 2. New Insights on Pediatric *H. pylori* Infection

*H. pylori* is a well-defined microaerophilic, Gram-negative bacterium that usually colonizes the human gastric mucosa during childhood and it might result in long-life inflammation and subsequent development of a wide spectrum of gastropathies like chronic gastritis, peptic ulcer disease and even certain types of malignant disorders [12,13]. Recent epidemiologic studies have reported a decreasing prevalence of pediatric *H. pylori* infection pointing out a significant difference between 25.6% in 2005 and 12.8% in 2017 [14]. Nevertheless, wide variations were reported among different populations in terms of prevalence mostly depending on the socio-economic status [7,8,14,15,16,17,18,19,20].

The clinical spectrum of this infection in children varies from asymptomatic to life-threatening conditions, such as peptic ulcer disease [21,22]. Nevertheless, the symptoms in children with *H. pylori* infection remain unspecific including abdominal pain, nausea, vomiting, heartburn [21,23], and thus the most recent recommendation of the European Society for Paediatric Gastroenterology, Hepatology and Nutrition (ESPGHAN) and the North American Society for Paediatric Gastroenterology, Hepatology and Nutrition (NASPGHAN) is to perform the necessary investigation for identifying the underlying cause of the symptoms and not only for assessing the presence of this infection [24]. A wide spectrum of extra-digestive conditions were proven to be associated to this infection, among which cardiovascular diseases, stroke, anemia, idiopathic thrombocytopenic purpura, eczema, rosacea, chronic hives, diabetes, thyroid disease or Alzheimer’s disease, which might be explained by the systemic inflammatory status identified even in pediatric patients diagnosed with *H. pylori*-positive gastritis [7,8]. Taking into account the severity of these aforementioned conditions and their long-life chronic pattern, the assessment of *H. pylori* related systemic inflammation should be a main goal in patients with this infection. The standard recommendation for diagnosing *H. pylori* infection implies only symptomatic children [24]. Thus, non-invasive tests are the most commonly used in daily practice in order to establish this diagnosis, but upper digestive endoscopy-based test definitely provide a more complete and accurate diagnosis.

The recent guidelines no longer recommend a ‘test and treat strategy’ in pediatric patients [24]. The standard therapy involves an association between a proton pump inhibitor and two antibiotics at high doses for 2 weeks [24]. Taking into consideration the differences between different countries in terms of exposure and common use of certain antibiotics, this therapy should be tailored for each population [10]. Thus, Silva et al. proved that tailored therapy associated with proton pump inhibitors in children results in an eradication rate of 97.8% [25]. In pediatric patients, therapy success is further hindered by poor compliance, inadequate dose or duration, inactivation of antimicrobial agents to low gastric pH or their inappropriate penetration into the gastric mucosa [10]. Another study that assessed the efficacy of sequential therapy on 40 symptomatic children using amoxicillin and lansoprazole for 5 days, followed by clarithromycin, metronidazole and lansoprazole for another 5 days proved that the eradication rate might reach up to 82.5% [26]. Potassium-competitive acid blockers, the most recent class of gastric acid suppressor, were assessed in high school students with *H. pylori* infection, in association with amoxicillin and clarithromycin, and the eradication was successful in 85.1% of the cases [20]. The supplementation of Lactobacillus might be beneficial in the eradication of *H. pylori* infection [27], but the studies on this topic remain controversial [10]. Thus, Eslami et al. pointed out that probiotics supplementation to triple therapy could increase the eradication rate and reduce the antibiotics side-effects [28]. Similar findings were encountered in pediatric patients according to a recent meta-analysis performed by Feng et al., who underlined that the effectiveness of triple therapy depends on specific probiotic supplementation [29].

In terms of pathophysiology the reports on children are scarce. Nonetheless, the relationship between microenvironmental factors, *H. pylori* virulence factors and immune activation are incontestable [10]. Specifically, the immune response mediated by toll-like receptors (TLRs) and microRNAs (miRNA) have been shown to play a role in the suppression or promotion of *H. pylori*-related gastropathies [11,30,31]. Thus, miRNA-155 is expressed by a wide-spectrum of immune cells in context of inflammatory response and it acts as an essential regulator of innate immune responses [30]. In pediatric patients, a study performed by Cortes-Marquez et al. concluded that the expression of certain types of miRNA might indicate the severity and the chronicity of the disease pointing out that the overexpression of miRNA-155 and miRNA-146a are associated with the persistence and outcome of *H. pylori* infection [31]. TLRs, which will be further detailed in another section, were also related to the persistence of this infection, playing a crucial role in gastric carcinogenesis [11]. Moreover, *H. pylori* binding avidity is definitely another sine qua non condition for the severity and persistence of this infection since the strains identified in children with peptic ulcer disease were shown to express a higher binding avidity as compared to those encountered in pediatric patients with non-ulcer dyspeptic symptoms [24]. Aside from the ability of this bacterium to bind and adhere to the gastric mucosa, other virulence factors were reported to be involved in promoting its persistence such as *cagA*, *vacA*, *babA2*, *homB* and *oipA* genes [10]. CagA is probably the most studied virulence factor and it is involved particularly in the development of both gastric and extra-digestive manifestations associated to *H. pylori* infection [32]. Moreover, the genera of *H. pylori* identified in children with peptic ulcer disease were shown to express a higher binding avidity as compared to those encountered in pediatric patients with non-ulcer dyspeptic symptoms [33]. Therefore, the complex interaction between all these factors related either to *H. pylori* itself or host-defense mechanisms definitely require further attention in order to delineate their precise role in children.

## 3. Innate Immunity and Gastric Microbiota—The ‘Yin and Yang’ of *H. pylori* Infection

Human homeostasis is defined by the proper relationship between microbiota and host’s immune responses [34]. The puzzle of gastric microbiota is still far to be elucidated. It is true that gastric microenvironment owns less hospitable living conditions for microorganism such as the proteolytic activity of the gastric juice, low pH, bile acids reflux, the synthesis of nitric oxide from salivary nitrate expressing antimicrobial properties; but still the presence of microorganisms in the gastric juice was identified 150 years before the description of *H. pylori* [35,36] destroying the hypothesis that stomach is a sterile environment. Further studies proved that bacteria are not the single microorganisms living in the stomach, identifying also different classes of fungi from the gastric samples [37]. *H. pylori* infection has a major impact on both gastric and colonic microbiota and several studies have assessed the interaction between this infection and the diversity of bacteria colonizing the gastrointestinal tract.

### 3.1. Gastric Microenvironment among Adults

Further studies revealed that gastric microbiota is diverse and depends on both host’s underlying gastropathy and external factors, such as geographic area. A very recent meta-analysis that assessed 36 studies on this topic pointed out that *Prevotella* and *Streptococcus* were the most frequently identified in the gastric samples, followed by Neisseria reported in 25 studies, as well as *Veillonella*, *Haemophilus* and *Fusobacterium*, identified in 24 of these studies [37]. Out of the 266 bacterial genera identified in these 36 studies, the majority were extremely rare or might even be artefacts as a result of bioinformatic processing or technical errors of sequencing [37]. The information regarding gastric microbiota in healthy individuals is limited since most of the studies focused on assessing patients with either *H. pylori* related gastropathies or gastric cancer. Nevertheless, 6 bacterial genera were reported in 88 healthy subjects: *Prevotella*, *Streptococcus*, *Megasphaerae*, *Oribacterium*, *Capnocutophaga* and *Propionibacterium* [37,38,39,40]. Another well-documented fact is that a wide diversity of gastric microbiota is encountered in healthy uncolonized subjects in comparison to *H. pylori* colonization, when studies reported both a lower abundance of other bacteria and a decrease in microbial diversity [41]. Therefore, we might state that *H. pylori* expresses a negative impact on both abundance and diversity of gastric bacterial community.

### 3.2. Gastric Microenvironment in H. pylori-Negative/-Positive Pediatric Subjects

In terms of pediatric patients, scarce information is available regarding the composition of gastric flora. A recent study performed on 346 children with symptoms suggesting *H. pylori* infection that aimed to assessed both *H. pylori* and non-*H. pylori* strains, pointed out 114 *H. pylori* positive cases and identified 366 non-*H. pylori* genera from 189 gastric biopsy specimens, 247 Gram-positive and 119 Gram-negative [42]. Moreover, the authors pointed out the following resistance rates for *H. pylori* strains: 86.4% for metronidazole, 36.4% for clarithromycin, 31.8% for levofloxacin, and 22.7% for both tetracycline and amoxicillin, as well as 10 multidrug-resistant strains [42]. Non-*H. pylori* bacteria identified in this pediatric cohort presented generally higher resistance rates to the same antibiotics: 94.8% for metronidazole, 46.7% for clarithromycin, 42.6% for amoxicillin, 26.2% in case of tetracycline and 15.3% for levofloxacin. Moreover, 63 isolates were found to be multidrug-resistant [42]. The presence of non-*H. pylori* bacteria within the gastric ecosystem is particularly important since they might persistently act as an antigenic stimulus or establish a partnership with *H. pylori* in order to augment the subsequent inflammatory [43]. Therefore, the gastric microbiota composition in children with *H. pylori* infection is dominated by *Streptoccocus*, *Neisseria*, *Rothia* and *Staphylococcus* [42]. The same study pointed out that several urease-positive non-*H. pylori* bacteria were present in pediatric gastric biopsy samples even in the absence of *H. pylori*, such as Staphylococcus aureus and epidermidis, *Pseudomonas plecoglossicida*, *Acinetobacter johnsonii*, *Neisseria mucosa*, *Neisseria flavescens*, *Neisseria meningitidis*, *Neisseria perflava*, *Rothia mucilaginosa*, and *Micrococcus luteus* [42]. Moreover, *Staphylococcus haemolyticus*, *Staphylococcus epidermidis*, *Neisseria mucosa*, *Micrococcus luteus*, *Rothia dentocariosa*, and *Actinomyces naeslundii* are well-known for their nitrate-reducing function with further implication in carcinogenesis due to the accumulation of nitrite and N-nitroso compounds in the gastric microenvironment [44]. Thus, we might hypothesize that gastric carcinogenesis has its origin in childhood when perturbation of stomach microecology homeostasis occurs.

Highly sensitive DNA-based detection methods suggest there may be many *H. pylori* carriers. This situation is particularly found in pediatric subjects since as we already mentioned the infection is usually acquired during childhood although they are not deemed always to develop the disease. Thus, a study performed on Spanish children proved that 17 of the children enrolled in this study were detected with a low abundance of *H. pylori*, carrying an average abundance of 0.45% within the gastric microbiota [45]. Moreover, a Chinese study revealed that five out of six individuals that were initially deemed *H. pylori* negative were found to be healthy carriers after sequencing-based methods, with a low abundance varying between 0.04–0.67% [46]. Therefore, we might reconsider defining the presence of *H. pylori* as an infection and instead use colonization for a better approach of this topic since *H. pylori* might be considered a ‘core member’ or, according to some authors, the only ‘true resident’ of the gastric microbiota [37].

Studies performed on mice pointed out that gastric commensal bacteria have the ability to accelerate the inflammation caused by *H. pylori*, increasing the risk of carcinogenesis [47]. Concerning this topic, children diagnosed with *H. pylori* infection were proven to harbor more diverse gastric bacterial communities when compared to infected adults [48]. The authors pointed out that infected children harbored lower abundance of *Firmicutes* and increased abundance of *Proteobacteria* along with higher proportions of *Gammaproteobacteria* in comparison to infected adult patients [48]. Regarding the genus level, *Rothia* was found to be less abundant in *H. pylori* positive children, while they harbored increased abundance of *Haemophilus*, *Neisseria* and an unidentified genus in *Neisseriaceae* family [48]. Thus, the assessment of changes in gastric commensal microbial community associated to *H. pylori* infection in children might be a cornerstone for the development of preventive and therapeutic strategies in order to reduce the persistence of this infection during adulthood and decrease the risk for long-term complications.

The interrelation between gastric commensal bacteria and age is undoubtable, and gastric microbiota might play a dichotomous role in promoting or suppressing *H. pylori*-related gastropathy.

### 3.3. Gastric Microenvironment in H. pylori-Negative/-Positive Adult Subjects

It is a well-established fact that *H. pylori* is the most common inhabitant of the gastric environment, but another 6 genera were identified in patients with *H. pylori* associated disorders: *Veillonella*, *Neisseria*, *Fusobacterium*, *Prevotella*, *Streptococcus* and *Haemophilus* [37]. Most of these bacteria were identified also in other parts of the gastrointestinal tract, such as oral cavity, small or large bowel, and they adjusted their ability to survive the gastric environment. Therefore, a potential hypothesis is that *H. pylori* is influenced and influences the gastric microbial community, although when present it tends to predominate and the patient’s final outcome depends on the network of coexisting bacteria [49]. This negative impact of *H. pylori* on the gastric microbiota is reversible according to Li et al., who proved that once eradicated, the gastric microbiota diversity can reach the levels of healthy uncolonized individuals [50]. The reciprocal relationship between *H. pylori* and gastric microbiota is therefore a decisive cornerstone for gastric carcinogenesis, and especially non-cardia adenocarcinomas. Bacterial virulence represented by certain allotypes, i.e., CagA and VacA is one of the major factors involved in gastric carcinogenesis. Recent studies hypothesized that the eradication of this infection might not only prevent gastric cancer, but also minimize the risk of metachronous cancer in patients treated for early gastric cancer [51]. In terms of gastric carcinogenesis there are still certain variables that require further studies, such as the role of other bacteria belonging to the gastric microbiome since studies revealed a wide variety of cohabitants in conditions of hypo/achlorhydria along with *H. pylori* that also increase the risk for carcinogenesis due to their ability of nitrosamine forming with a well-documented oncogenic potential [52]. Thus, we hypothesize that an abnormal gastric microenvironment harbors an abnormal microbiota that additionally contributes to the *H. pylori* oncogenic potential.

In terms of *H. pylori*, multiple hypotheses have been proposed in order to explain the co-occurrence or mutual exclusion of microorganisms, such as the secretion of 20-amino-acid-long cecropin-like antibacterial peptides leading to a pro-inflammatory response and subsequent local acidosis; gastrin release; initial reduction of gastric pH in the acute phase resulting in an abundance of *H. pylori* that tends to predominate the gastric environment; a further increase of gastric pH associated to *H. pylori* chronic infection, which has exactly the opposite effect resulting in a microbial bloom that further inhibits the growth of *H. pylori* [49,53,54,55]. Moreover, the increase in gastric pH as a result of *H. pylori* chronic infection is a sign of atrophic gastritis that might influence itself the growth of certain microbes within the gastric environment. Thus, a study performed on patients with autoimmune chronic gastritis identified Gemella and Bosea as the main bacteria genera colonizing the stomachs of these patients [56], although the reports remain controversial. *H. pylori* influences and is influenced by the gastric microbial community.

### 3.4. Gastric microenvironment in H. pylori-Negative/-Positive Subjects Treated with Proton Pump Inhibitors or Probiotics

Certain drugs that are also used in *H. pylori* eradication regimens were proved to impact the diversity and abundance of gastric microbial community. Proton pump inhibitors (PPIs), due to their ability to increase the gastric pH, definitely result in considerable changes of gastric microbiota composition. Thus, a study performed on Israeli patients before and after PPIs administration proved that major changes occurred in gastric microbiota after 8 weeks of treatment consisting of a significant decrease in *Flavobacteriaceae*, *Moraxellaceae*, *Comamonadaceae* and *Methylobacteriaceae*, along with an increase in *Erysipelotrichaceae* and a family from the order *Clostridiales* [57]. Another study proved that normal healthy subject harbor similar gastric bacterial profiles to patients undergoing PPIs treatment, but underlined differences in terms of abundance encountering *Prevotellaceae* as the most abundant bacterial family in control group, followed by *Streptococcaceae*, *Paraprevotellaceae* and *Fusobacteriaceae*; while in PPIs group, the most abundant was *Streptococcaceae*, followed by *Prevotellaceae*, *Campylobacteraceae*, and *Leptotrichiaceae* [56]. Thus, PPIs clearly impact the composition, diversity and abundance of gastric bacterial community, but further studies on larger cohorts are required to elucidate the consequences of these changes.

Another class of therapeutic agents that has an effect on gastric microbiota are probiotics, but this topic is far to be understood. Nevertheless, a study that assessed *Lactobacillus gasseri* OLL2716 in patients with functional dyspepsia and healthy controls underlined that this therapy has the ability to change gastric fluid microbiota composition in these patients resulting in similar composition to that of healthy volunteers [58]. Thus, probiotics supplementation in *H. pylori*-positive patients undergoing standard regimens might be useful for an effective eradication.

### 3.5. The Trialogue between H. pylori, Gastric Microbiota and Immune System

A less explored side of this balance between *H. pylori*, gastric microbiota and immune system is represented by the effects of gastric bacterial community on immune responses with further crucial impact on *H. pylori* persistence and its associated gastropathies. Gastric microbiome could be defined as a complex genomic content including microbial communities, host epithelium and host’s immune system elements [59]. Moreover, similar to the statements of Nasr et al., we hypothesize that when dysbiosis occurs, i.e., changes in the number or diversity of gastric bacterial community, the commensal gastric bacteria become ‘pathobionts’, and along with dietary carcinogenic factors or other environmental factors, they play a key role in promoting carcinogenesis [59]. Thus, dysbiosis enhances growth and invasion of different pathogenic bacteria, further impairing the balance between the immune system and mucosal barrier. Pathogen recognition receptors (PRRs) are one of the main components of the immune system and they are trained in recognizing potentially pathogenic bacteria from harmless commensal community. Toll-like receptors (TLRs) represent an essential class of PRRs and they were identified on the surface of macrophages and dendritic cells [59] (Table 1).

Macrophages are well-known to accumulate within the gastric tissue as a response to *H. pylori* infection and the increase in their number is closely related to the disease severity and infection persistence [81,82]. Except of its chemotactic effect, *H. pylori* owns the ability to activate macrophages and induce the synthesis of interleukins (IL) 1, 6 and 8, but also tumor necrosis factor alpha (TNF α), MIP-1 alpha and GRO-alpha, which further express a positive feedback on the recruitment and activation of additional macrophages and T cells [83,84]. In addition to their function of expressing TLRs, macrophages also secrete products with a negative impact on the gastric tissue in the setting of *H. pylori* infection, among which matrix metalloproteinases 9 and 2, which were proved to be elevated in patients with this infection, being involved in the destruction of gastric tissue [84,85]. It was proved that macrophages and monocytes act as essential coordinators of immune responses in the setting of *H. pylori* activating adaptive immunity through IL 12 synthesis, which will further stimulate Th1 cytokines such as IFN γ [86]. Moreover, *H. pylori* plays a key role in favoring the migration of polymorphonuclear cells to the gastric mucosa via its virulence factor called *H. pylori* neutrophil-activating protein (HP-NAP) [86]. Another crucial role of this protein is the modulation of adaptive immunity by promoting the production of Th1 with subsequent increased synthesis of IFN γ, TNF α, and increasing the cytolytic activity of these cells [86]. Thus, we might state that both macrophages and neutrophils form a partnership with *H. pylori* in modulating the activity of Th1 cells with a positive feedback on Th1 cytokines production. Th1 lymphocyte response is also positively influenced by gastric epithelial cells that act as antigen presenting cells activating CD4^+^ Th cells [86]. Other T cell populations are involved in the immune response modulation to *H. pylori* such as Th17, which mainly produce IL 17A [87]. Furthermore, IL 17A promotes the expression of cytokines that act as chemoattractant for neutrophils from epithelial cells [88]. Aside from IL 17A, Th17 also secrete IL 17F, IL 21 and IL 22, which contribute to the recruitment of inflammatory cells [87]. Thus, Il 17/Th17 response promotes the persistence of the bacterium resulting in a chronic inflammatory process [86].

The activation of TLRs signaling pathways triggers the synthesis of inflammatory cytokines, chemokines, costimulatory molecules and antigen-presenting molecules [89]. Two main pathways were characterized in the activation of these receptors: myeloid differentiation primary response-88 (MyD88) and TIR-domain-containing adapter-inducing interferon β (TRIF), which result in the expression of multiple cytokines, among which IL 1 β and 6, as well as interferons gamma (IFN γ) and gamma-induced protein 10 [90]. Moreover, the expression of MyD88 in macrophages was proved to be crucial for the induction of proinflammatory cytokines in the setting of *H. pylori* infection such as IL 6, 1β, 10 and 12 [63,64,91]. In addition, IL 8 was also proved to be overexpressed in the presence of *H. pylori* according to a study performed on cultured gastric epithelial cells as a result of both TLR 2 and 4 activation by *H. pylori* heat shock protein 60, and subsequent augmentation of nuclear factor-κB activity [66]. Studies performed on mice proved that both TLR 2 and 4 are essential for these responses in macrophages [63,64]. It seems that these two TLRs interact and they are both involved in the recognition of *H. pylori* lipopolysaccharide, and that TLR2 also triggers a Th1 immune response since it binds to *H. pylori* neutrophil-activating protein [11,92]. It is also worth mentioning that dysbiosis-associated chronic inflammatory diseases, such as *H. pylori*-induced chronic gastritis result in an alteration of TLRs expression and subsequent downstream signaling. In normal settings, these TLRs have the ability to initiate fast and effective inflammatory responses for hindering microbial invasion [93], whereas *H. pylori* is well-documented to impair their normal functioning and to escape their recognition for establishing its persistence within the gastric tissue [11].

An association between TLR 4 signaling and T regulatory cells was underlined in mice, pointing out that the suppression of this signaling pathway results in an overcolonization of *H. pylori* due to an increase in T regulatory cells [68]. Additionally, it was emphasized that certain commensal intestinal bacteria have the ability to induce the differentiation of these T regulatory cells locally by elaborating several products like short chain fatty acids [94,95], and capsular polysaccharides [96,97]. The study of Brawner et al. indicated that the altered gastric microbiota due to *H. pylori* infection is involved in increasing the gastric T regulatory cell response in children pointing out that *H. pylori* infected pediatric patients with subsequent altered gastric microbiota have significantly increased levels of IL 10 as compared to healthy children and *H. pylori*-positive adults due to the enhanced FOXP3 response, a protein involved in immune response [48]. The same pattern was observed for transforming growth factor (TGF) β, another anti-inflammatory cytokine, the expression of which was found to be elevated in children with *H. pylori* infection [48] since this infection contributes to both induction and maintenance of TGF β expression [98,99]. Based on all these facts, the relationship between altered gastric microbiota and immune responses defined by mucosal T regulatory cells in children is incontestable. A potential mechanism for the long-term persistence of *H. pylori* infection that commonly occurs during childhood is based on the development of T regulatory-mediated tolerance to this bacterium, and further suppression of the effector T-cell responses [48]. TLR 5 is involved in the recognition of bacterial flagellin, through the p38 MAP kinase signaling pathway [11]. Moreover, this TLR together with TLR 2 were proven to influence the strains of *H. pylori* that carry the CagA pathogenicity island using coculturing of monocytic THP-1 cells [70]. TLR 9 recognizes bacterial DNA resulting in proinflammatory responses as it was proved on murine models [72]. Contrariwise, another study performed on mice showed that this TLR might also act as a suppressor for *H. pylori* infection during the acute phase of this infection since it is able to promote anti-inflammatory signaling [74]. Thus, the dichotomous role of TLR 9 seems to be influenced by the gastric microenvironment [4], emphasizing once more the essential role of gastric microbial community in immune responses even in pediatric patients.

Defensins, defined as short proteins belong to the family of mammalian antimicrobial peptides (AMPs) and they are known for their antimicrobial activity not only against bacteria, but also viruses, fungi and protozoa [100,101]. The expression of β-defensin 2 was found to be increased in the presence of bacteria [102]. Additionally, *H. pylori* induced gastritis was proved to be associated with considerable increase in β-defensin 2 as compared to non-*H. pylori* gastritis [103]. Innate host defense definitely owns a crucial role in eliminating gastric injurious factors since β-defensins were revealed to be increased in the setting of bacterial inflammation [104]. AMPs have a major influence on shaping the formation of gastrointestinal microbiota [34]. Nevertheless, this relationship is reciprocal since it is a well-documented fact that gastrointestinal microbiota regulates the secretion of defensins [34]. Multiple factors contribute to the modulation of β-defensins in the setting of *H. pylori* infection involving strain-dependent mechanisms and genetical factors, such as Cag pathogenicity island [105]. Gastric microbiota is closely related to gut commensal bacteria and in fact each part of the gastrointestinal microbiota is a small puzzle piece that functions optimally only when attached to each other. Therefore, gut microbiota is involved in the regulation of defensins production with a fast and effective role in deactivating or destroying microorganisms [106]. Furthermore, for a proper and effective protection against pathogens invasion, defensins must form a partnership with certain mediators, like cytokines, chemokines, supplements, antimicrobial proteins or cellular elements [107]. The crosstalk between *H. pylori* and immune system in pediatric patients is far to be fully understood, but both innate and adaptive immune response play a key role in the host’s response to this infection and might represent future targets for individualized therapies.

## 4. Conclusions

Gastric homeostasis depends mostly on the appropriate interplay between the local commensal bacterial community and host’s immune system. *H. pylori*, the most common bacterial infection worldwide usually acquired during childhood, is influenced and influences gastric microbiota. Moreover, host’s immune system shapes the development of gastric microbiota and plays a decisive role in promoting or suppressing *H. pylori* infection. Nevertheless, *H. pylori*, gastric microbiota and host’s immune responses form a complex puzzle that is far to be properly assembled requiring further studies especially in pediatric patients taking into account the age-related differences reported so far.

## Figures and Tables

**Table 1:** The role of TLRs.

No.	Type of TLR	Components of *H. pylori* Activate	Place	Results/Effects	Observations
1.	**TLR 1**	*H. pylori* surface	Cell surface (Kawai et al., 2011) [60] Cell surface of bacteria, fungi, parasites and Viruses (Kawai et al., 2009) [61]	Binding partners for TLR2, aiding in its ability to recognize different ligands (Oliveira-Nascimento et al., 2012) [62] With TLR2 recognize a wide range of pathogen-associated molecular patterns (PAMPs) derived from various pathogens, ranging from bacteria, fungi, parasites and viruses (Kawai et al., 2009) [61]	-
2.	**TLR 2**	binding to *H. pylori* neutrophil-activating protein (Rad et al., 2007; Obonyo et al., 2007; Takeda et al., 2005) [63,64,65] nuclear factor-*κ*B activity (Takenaka et al., 2004) [66] TLR2/MyD88 signaling (Echizen et al., 2016) [67]	cell surface (Kawai et al., 2011) [60] macrophages culture gastric cells monocyte cell surface of bacteria, fungi, parasites and viruses (Kawai et al., 2009) [61]	recognition of *H. pylori* lipopolysaccharide triggers a Th1 immune response stimulate IL 8 influence the strains of *H. pylori* that carry the CagA pathogenicity island using coculturing of monocytic THP-1 cells pathogenesis of gastric cancer worldwide recognize a wide range of pathogen-associated molecular patterns (PAMPs) derived from various pathogens, ranging from bacteria, fungi, parasites and viruses (Kawai et al., 2009) [61]	studies on mice study on mice
3.	**TLR 3**	*H. pylori* vesicles	intracellular vesicles- endoplasmatic reticulum (Kawai et al., 2011) [60] *dendritic cells* (Kawai et al., 2009) [61]	in the recognition of microbial nucleic acids (Kawai et al., 2011) [60]	-
4.	**TLR 4**	binding to *H. pylori* neutrophil-activating protein (Rad et al., 2007; Obonyo et al., 2007) [63,64] nuclear factor-*κ*B activity (Takenaka et al., 2004) [66]	*MD-2 cell surface* (Kawai et al., 2011) [60] macrophages apical and basolateral levels of the membrane compartiment membrane of Gram-negative bacteria, (Kawai et al., 2009) [61]	recognition of *H. pylori* lipopolysaccharide stimulate IL 8 modify the lipid A core of its LPS, representing a possible ligand for the immune complex TLR4-MD2, in order to avoid TLR4 recognition (Varga et al., 2017) [4] inhibiting CD25 expression could decrease T regulatory cells and, as a result, could suppress *H. pylori* colonization (Gong et al., 2016) [68] a potent immunostimulatory molecule and causes septic shock (Kawai et al., 2009) [61]	studies on mice
5.	**TLR 5**	p38 MAP kinase signaling pathway (Schmausser et al., 2005) [69]	*cell surface* (intestinal cells, kidney & bladder) -monocyte (Kawai et al., 2011) [60], (Kawai et al., 2009) [61]	recognition of bacterial flagellin [70] influence the strains of *H. pylori* that carry the CagA pathogenicity island using coculturing of monocytic THP-1 cells risk for gastric carcinoma (Xu et al., 2017) [71] recognize flagellin, a protein component of bacterial flagella (Kawai et al., 2009) [61]	-
6.	**TLR 6**	are expressed exclusively on the cell surface (Kawai et al., 2009) [61]	*cell surface* (Kawai et al., 2011) [60] *cell surface* of bacteria, fungi, parasites and viruses (Kawai et al., 2009) [61]	binding partners for TLR2, aiding in its ability to recognize different ligands (Oliveira-Nascimentoet al, 2012) [62] recognize microbial membrane components such as lipids, lipoproteins and proteins (Kawai et al., 2009) [61] with TLR2 recognize a wide range of pathogen-associated molecular patterns (PAMPs) derived from various pathogens, ranging from bacteria, fungi, parasites and viruses (Kawai et al., 2009) [61]	-
7.	**TLR 7**	*H. pylori* RNA	intracellular vesicles —endoplasmic reticulum (Kawai et al., 2011) [60] plasmacytoid dendritic cells (Kawai et al., 2009) [61]	induces pro-inflammatory cytokines in an MyD88-dependent manner	-
8.	**TLR 8**	*H. pylori* RNA	intracellular vesicles—endoplasmatic reticulum (Kawai et al., 2011) [60]	induces pro-inflammatory cytokines in an MyD88-dependent manner (Rad et al., 2009) [72] stimulation of T regulatory cells hinders their inhibitory action (Peng et al., 2005) [73]	-
9.	**TLR 9**	DNA of bacteria (Rad et al., 2009 [72] Suppression for *H. pylori* infection Otani et al., 2012 [74]	intracellular vesicles—endoplasmatic reticulum (Kawai et al., 2011) [60]	recognizes bacterial DNA resulting in proinflammatory responses suppresor for *H. Pylori* infection during the acute phase of this infection —promoting the anti-inflammatory signaling (Otani et al., 2012) [74] promote pro-inflammatory cascades, and eventually result in the development of gastric cancer (Schmausser et al., 2004) [75]	Murine model mice
10.	**TLR 10**	a functional receptor involved in innate immune response triggered by *H. pylori* (Nagashim et al., 2015) [76]	*B Cells and Plasmacytoid Dendritic Cells* (Hasan et al., 2005) [77]	*TLR10* rs10004195 gene polymorphisms are associated with a higher risk of gastric cancer Ravishankar Ram et al., 2015) [78] Activates Gene Transcription through MyD88(Hasan et al., 2005) [77]	TLR10 is not functional in mice because of a retrovirus insertion (Kawai et al., 2009) [61]
11.	**TLR 11**	innate sensor for the Toxoplasma protein profilin (Pifer et al., 2010) [79]	intracellular vesicles—endoplasmatic reticulum (Kawai et al., 2011) [60] & dendritic cells, kidney & bladder (Pifer et al., 2010) [79], (Kawai et al., 2009) [61]	regulates the activation of dendritic cells in response to Toxoplasma gondii profilin and parasitic infection in vivo (Pifer et al., 2010) [79]	-
12.	**TLR 12**	reticulum-resident protein UNC93B1 (Pifer et al., 2010) [79]	intracellular vesicles—endoplasmatic reticulum (Kawai et al., 2011) [60] & dendritic cells (Pifer et al., 2010) [79]	activation of dendritic cells in response to Toxoplasma gondii profilin and parasitic infection in vivo (Pifer et al., 2010) [79]	-
13.	**TLR 13**	are expressed intracellularly, (Blasius et al., 2010) [80]	endoplasmic reticulum, multivesicular bodies, and lysosomes (Blasius et al., 2010) [80]	TLR13 of mice are expressed intracellularly within the endoplasmic reticulum (ER) trafficking of Intracellular TLRs (Blasius et al., 2010) [80]	mice
